# Religious coping in time of covid 19 in tunisia

**DOI:** 10.1192/j.eurpsy.2021.761

**Published:** 2021-08-13

**Authors:** S. Ajmi, S. Hentati, R. Masmoudi, R. Sellami, I. Feki, J. Masmoudi

**Affiliations:** 1 Psychiatrie A, hospital university Hedi Chaker, sfax, Tunisia; 2 Psychiatry A, hedi chaker hospital, Sfax, Tunisia; 3 Psychiatrie “a” Department, Hedi Chaker Hospital University -Sfax - Tunisia, sfax, Tunisia

**Keywords:** COVID19, religious coping, lockdown

## Abstract

**Introduction:**

Religion belongs among well-documented coping strategies, through which one can understand and deal with stressors.

**Objectives:**

The aim of this study was to examine religious coping responses face to the outbreak of COVID-19 pandemic among Tunisian people.

**Methods:**

The survey was conducted using the online anonymous questionnaires and distributed through social networks from 24 April to 23 May 2020. It included sociodemographic questions, participants’ experience of SARS-CoV-2related stressful events and the frequency of religious practice during the COVID-19 pandemic. The Brief RCOPE was used to assess religious coping.

**Results:**

Our study included 80 participants: 71.3%female and 42.5%married. The mean age of the participants was 29.30 years (SD = 8.72). The religion of all participants was Islam, and 72.5% of them had religious practices. Participants reported much lower levels of negative religious coping than positive religious coping (5% versus 37.5%). There were no significant differences in religious coping activities as a function of gender (p=0.180, p= 0.192). Significant relationships were found only for demographic variables: level of education with Higher-educated reported more PRC (p=0.002). Having a family member with a suspected or confirmed infection was correlated with PRC (p=0.016).Concern with becoming infected or having a friend with a suspected or confirmed infection did not correlate with any coping strategy (p=0.112; p=0.489). No correlation was found between religious commitment and religious coping (p=0.897; p=0.504) however increasing religious activity during this pandemic was correlated with PRC (p=0.013).

**Conclusions:**

Our findings suggest that lockdown experience is associated with higher use of NRC strategies.

